# Exploring the Mechanisms of Neuronal Protection by Glial Cell Line-Derived Neurotrophic Factor in Autism Spectrum Disorder

**DOI:** 10.7759/cureus.70913

**Published:** 2024-10-05

**Authors:** Sarwat Ali Raja, Amna Batool, Maryum Sana, Hafiz Muhammad Haseeb Khaliq, Faiza Choudhry, Durga Devi

**Affiliations:** 1 Pharmacy, Yashfeen Education College of Pharmacy & Allied Health, Bhawalpur, PAK; 2 Surgery, Fatima Memorial Hospital, Lahore, PAK; 3 Nursing, Akhtar Saeed College of Nursing, Lahore, PAK; 4 College of Nursing, Akhtar Saeed Medical and Dental College, Lahore, PAK; 5 Molecular Diagnostics and Pathology, University of Health Sciences, Lahore, PAK; 6 Medicine and Surgery, People’s University of Medical and Health Sciences, Middlesex, USA; 7 Pathology, Liaquat University of Medical and Health Sciences, Jamshoro, PAK

**Keywords:** autism spectrum disorder (asd), correlates of cognitive impairment, gdnf, neurobiology, neuroprotection

## Abstract

Background: A complicated neurological disease known as autism spectrum disorder (ASD) is typified by issues with social interaction, communication, and repetitive behavior. The neural protective mechanisms in ASD are thought to be influenced by genetic variables, including the expression of neurotrophic genes such as glial cell line-derived neurotrophic factor (GDNF).

Objective: The aim was to examine the relationship between neuronal protection and cognitive functioning by crosslinking GDNF gene expression and serum levels in individuals with relation to Mini-Mental State Examination (MMSE) scores in ASD patients.

Materials and methods: After getting study approval and informed consent of patients, this case-control study experimental study was conducted for six months between July 2023 and December 2023. The blood samples (5 ml each) were drawn from the study population (n = 140), including 100 ASD patients with a disease course of 30 months based on patients' reports data and 40 healthy controls from four major clinical and hospital settings in Lahore, Karachi, and Bahawalpur from Pakistan. The analytical procedures included nucleic acid extraction, primer design and optimization, and GDNF-targeted real-time quantitative polymerase chain reaction expression analysis. To measure cognitive and behavioral deficits, enzyme-linked immunosorbent assay-based serum GDNF levels (pg/ml) and MMSE scores were compared, concluding the neuronal protection potential of GDNF.

Results: In patients with ASD, lower serum levels of GDNF (9.371 ± 2.388 pg/ml) were linked to more severe behavioral and cognitive deficits confirmed by MMSE scores (13.6 ± 3.5) of ASD patients in comparison with the control group (27.1 ± 2.1). Healthy individuals showed higher relative gene fold expression (11.71) compared to the ASD patients (5.51).

Conclusion: There is a notable decrease in GDNF gene expression in people with ASD, which raises the possibility that GDNF is important for both cognitive performance and neuronal protection in these people. GDNF may be a useful biomarker for identifying ASD and comprehending its molecular causes, opening the door for focused treatment approaches.

## Introduction

Autism spectrum disorder (ASD) is a multifaceted neurodevelopmental disorder marked by repetitive behaviors, speech challenges, and issues with social interaction [1]. The pathophysiology of ASD is largely dependent on neurotrophic proteins and genetic variables, as recent research has shown. For this reason, studying genes such as glial cell line-derived neurotrophic factor (GDNF) is important [2]. The neurotrophic factor protein family, to which GDNF belongs, is well-known for having a substantial influence on the survival, differentiation, and maintenance of neurons. This component is essential to neurodevelopmental processes because it promotes neuronal growth and survival throughout childhood and adolescence [3]. GDNF's neuroprotective qualities are particularly crucial in halting the neuronal degeneration of neurons seen in several neurological disorders, including ASD [4].

Reduced GDNF levels have been linked to higher levels of inflammation and oxidative stress, both of which are often worse in people with ASD. These elements may exacerbate existing neural damage and impair cognition [5]. Numerous potential genes linked to ASD have been found in genome-association studies. Of these, GDNF has emerged as a new gene of interest because of its regulatory and neuroprotective properties [6]. The neurophysiological functions of GDNF include enhancing neurogenesis, synaptic plasticity, and general brain function, all of which are essential for preserving behavioral and cognitive health in people with ASD [7]. The roles of the neurotrophin family, which includes GDNF, BDNF, NT-4/5, and NT-3, in maintaining and promoting neuronal survival have been well investigated. Particularly, GDNF interacts with receptors that are important for several neurophysiological processes necessary for brain health and function, including TrkA, TrkB, TrkC, and the low-affinity receptor p75NTR [8].

The Mini-Mental State Examination (MMSE; 30 mpoints) is a brief questionnaire that is widely used to assess cognitive impairment in clinical research. It assesses cognitive abilities like math, memory, and orientation and yields a numerical score that may be used to monitor improvements over time [9]. In our study, we attempt to establish a relationship between the degree of cognitive and behavioral deficits as determined by MMSE scores and the expression levels of GDNF, opening up new possibilities for early detection and focused treatment approaches.

## Materials and methods

A case-control study with a duration of six months was conducted based on 140 blood samples collected between July 2023 and December 2023. This study was based on 30 months of diagnostics history of 100 patients with ASD and 40 healthy controls, collected from four major hospital settings in Lahore, Karachi, and Bahawalpur, Pakistan (Yashfeen Medical Institute Bahawalpur, Fatima Memorial Hospital Lahore, Akhtar Saeed Hospital Lahore, and Peoples University of Medical and Health Sciences Middlesex), via consecutive sampling techniques. All authors contributed to the study's conception, design, and data acquisition in accordance with the predefined standard operating procedures (SOPs) for research conduct. The inclusion and exclusion criteria were determined before the study commenced, and informed consent forms, along with MMSE score sheets, were distributed to all participating institutes to ensure uniformity across the study. Inclusion criteria were age (>40 years), male and female, healthy controls, and confirmed cases of diabetes and autism. Exclusion criteria consisted of lack of clinical history, absence of diagnostic test records, refusal of informed consent, and cancer patients. MMSE scores were calculated based on 30 points to evaluate the patients' ability to carry out basic mathematical calculations, ability to recall events, and other aspects that pertain to the patient's orientation. All blood samples were transported under standard transport protocols to affiliated diagnostic laboratories for nucleic acid extraction and enzyme-linked immunosorbent assay (ELISA) analysis. Data acquisition for MMSE scores and serum GDNF levels was performed collaboratively. Data interpretation and analysis were conducted at respective institutes, with final approval of the accumulated summary given by all authors. Study approval no. 06/04/ERC-716-2023 was issued on June 6, 2023, at Liaquat University of Medical and Health Sciences, Jamshoro, Pakistan. Approvals from the respective institutes were also obtained before the study conductance

GDNF serum levels were measured by ELISA using a kit for quantitative determination (IHUADPNKTC # IH0556) according to the manufacturer’s protocol. A Qiagen blood kit (QIAamp#56604) was used to isolate genomic DNA from peripheral blood, and amplification was performed on Thermocycler (Bio-Rad-114) using PCR Master Mix (Thermo Fisher Kit# 4426518) in 40 µL of RNAse-free water containing 0.35 µM primers. The PCR conditions used were four minutes of initial denaturation at 95°C, one minute of denaturation at 94 °C, 15 seconds of annealing at 53°C, and one minute of extension at 72°C. The primers were designed on a serial cloner by using the consensus sequence of specific genes from the NCBI database, and then primer specificity or universality was checked by primer-BLAST or BLASTn, respectively. Sequences of the designed primers (forward and reversed) are shown in Table 1.

**Table 1:** Sequences of glial cell line-derived neurotrophic factor designed primers

Primer pair	Sequence (5/ to 3/)
Froward	TGCTGGCCTAATAGAGTGGC
Reverse	CTCAGCGCCATGGAAAATGT

One-way ANOVA was conducted to find variance among the samples with IBM SPSS Statistics for Windows, version 26.0 (released 2019, IBM Corp., Armonk, NY). A p-value of >0.05 was considered statistically significant.

## Results

In this case-control study, a total of 140 blood samples (61 male and 79 female) were taken for the investigation including 40 healthy controls. The average age of males and females were 52.4 and 56.1 years, respectively, with standard deviations as shown in Table 2. The number of cases of males and females was 61 and 79, respectively. All verified instances of autism with diabetes were evaluated using the MMSE and GDNF levels (pg/ml), as shown in Table 3.

**Table 2:** Demographics of the study population (n = 140)

Gender	Average age ± standard deviation	Cases (%)
Male	52.4 ± 8.5	61
Female	56.1 ± 4.6	79

**Table 3:** Gene expression analysis, serum levels, and Mini-Mental Score Examination scores of the study population (n = 140)

Variables	Autism patients (n = 100)	Healthy Controls (n = 40)	T-test (p-value)
Gene expression (pg/ml)	8.371 ± 2.388	15.371 ± 3.64	0.013
scores	13.6 ± 3.5	27.1 ± 2.1	0.008

**Figure 1:**
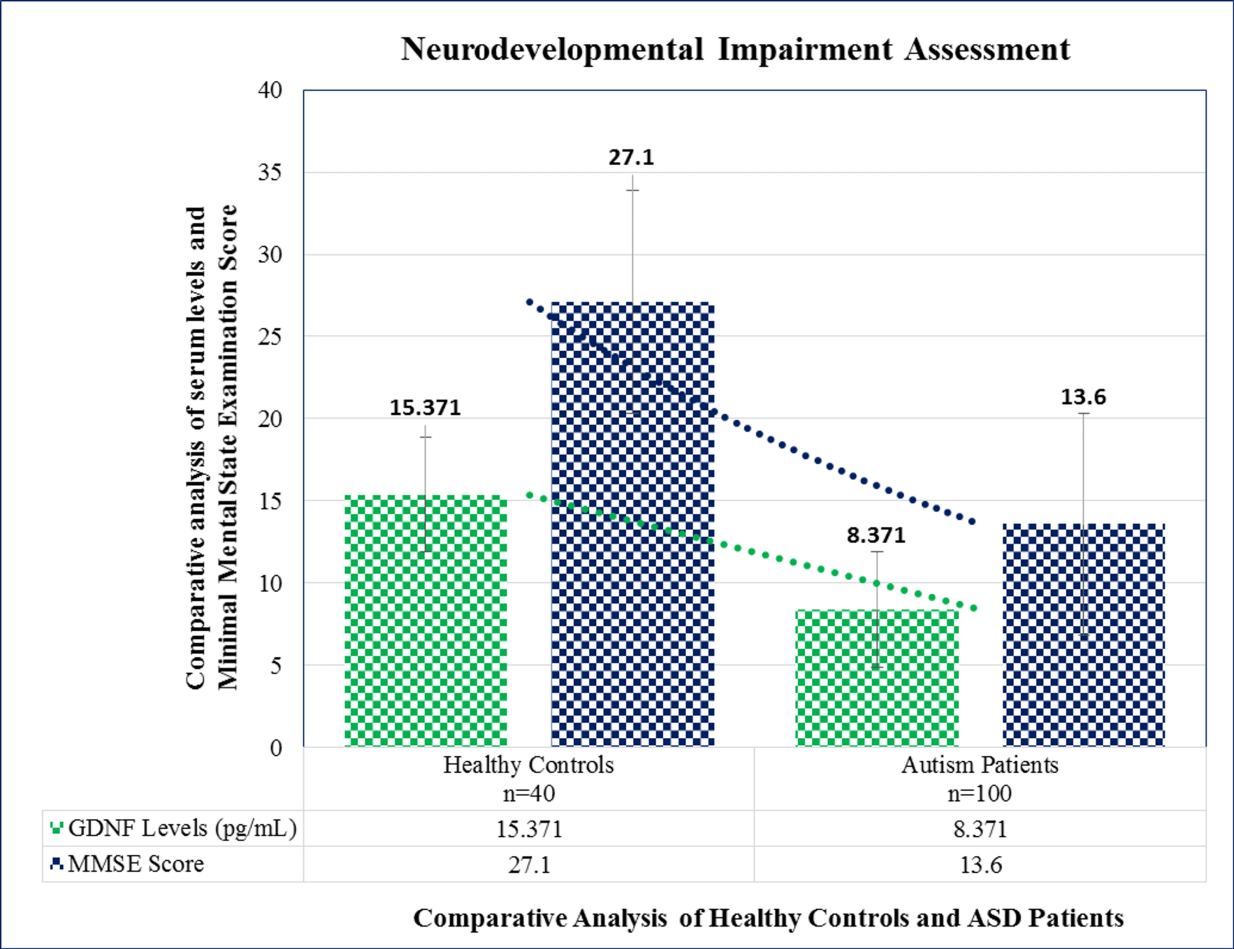
Comparative analysis of the ASD patients and MMSE score analysis In comparison to healthy individuals, autism patients showed decreased serum levels and low MMSE scoring, confirming the assessment for the phenomenon of cognitive impairment in autism. ASD: autism spectrum disorder, MMSE: Mini-Mental State Examination

**Figure 2:**
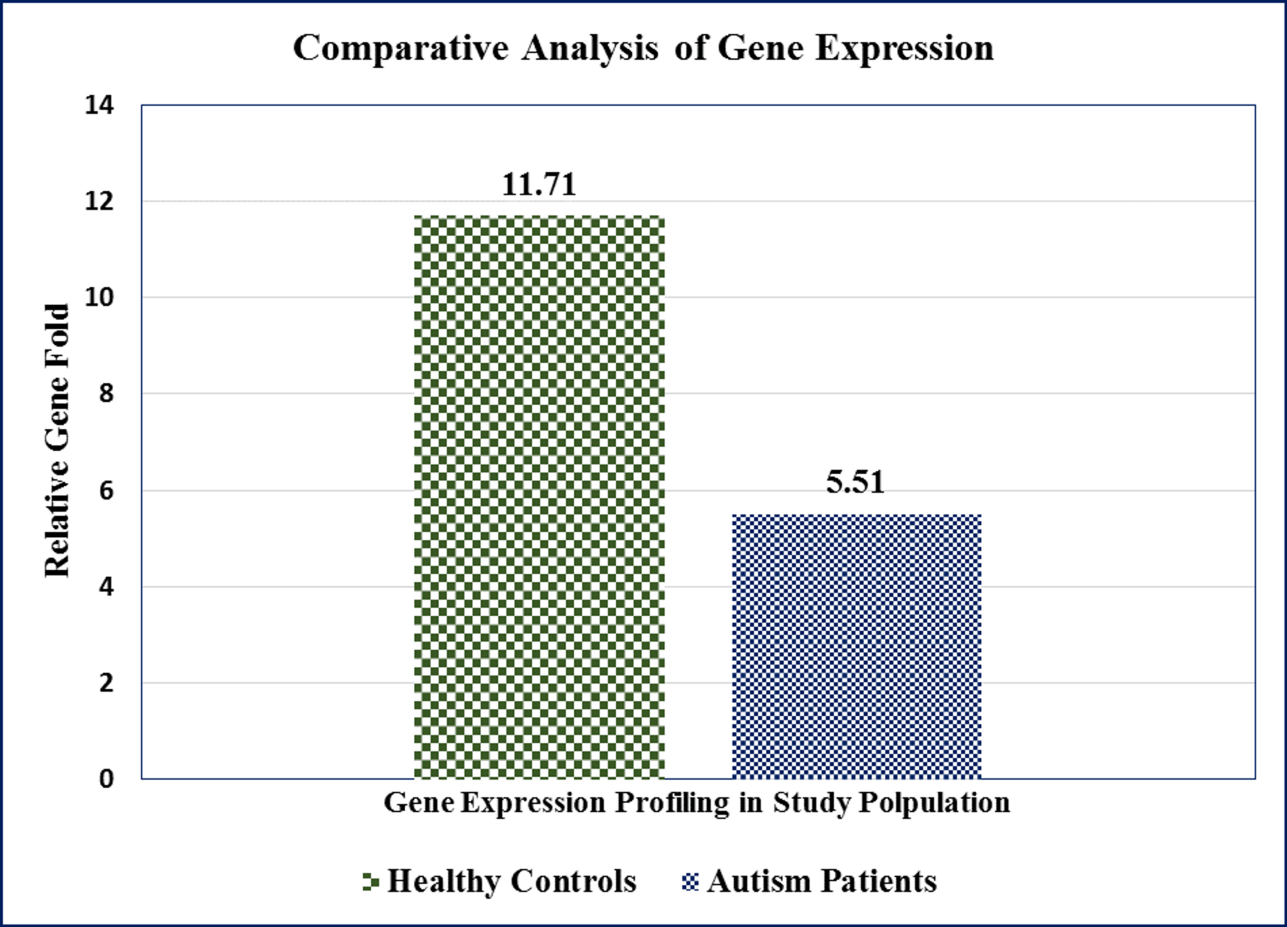
Relative gene fold change in healthy controls and autism cases. Our data showed that healthy individuals possess higher relative gene fold expression (11.71) compared to the autism patients (5.51).

The MMSE questionnaire was used to assess the mental state of the patients. The individuals were classified as having less or no cognitive impairment (24 or higher score) and severe cognitive impairment (0-15) based on their MMSE scores. The medical experts who created the exam scored the results based on the predetermined criteria that were included in the questionnaire. There was a direct relation seen between the MMSE scoring and GDNF levels between the healthy controls and ASD patients. Figure 1 illustrates how the severity-based scoring interpretation is determined, and Figure 2 illustrates the comparative analysis of relative gene fold expression:

## Discussion

Investigating the expression of GDNF genes provides important insights into putative neural defense mechanisms in the fight to comprehend and treat ASD. ASD causes problems in cognition, behavior, and social interaction by affecting several brain areas and neural networks [10]. A person’s likelihood of developing cognitive impairments rises with conditions that have been shown in literature as the main causes of neurodegenerative disorders and neurodevelopmental disorders like ASD, including oxidative stress and neuroinflammation. In particular, we revealed that GDNF gene abnormalities in our model worsen the condition by enhancing oxidative stress and neuroinflammatory processes, which have been indicated to predict a faster degradation in cognitive abilities in persons with ASD [11].

Similar findings have been reported in observations on the use of GDNF in neurodegenerative diseases where there is low expression of GDNF with a resulting bad neuroprotection extended by oxidative stress [12]. For example, examining neuroinflammation models, reported that a decrease in GDNF levels directly correlates with the synaptic abnormalities, thus proving the hypothesis that GDNF is neuroprotective. In the present study, we obtained a similar result with our RT-PCR findings showing that the expression of GDNF was significantly reduced in ASD and they emphasize the possibility that GDNF may serve as a molecular marker of ASD development. These outcomes accord with the study that supports the hypothesis that GDNF deficiency may lead to such neurodegenerative processes in Alzheimer’s disease: amyloid precursor proteins are retained in the Golgi apparatus due to insufficient GDNF levels [11,12].

Our result of under-expression also supports previous research such as the study conducted where the authors show that low levels of GDNF are associated with synapse dysregulation and neuronal shrinkage, both of which are characteristic neuropathological features in neurodevelopmental disorders [13]. The dramatic decrease in GDNF levels in our group of ASD patients further served to underscore the link between GDNP downregulation and the previously described MMSE scores that did not exceed 10 in the great majority of cases. This is in concordance with a study that revealed that patients with thinking and memorizing impairments associated with neuroinflammation register a similar fold decrease in GDNF expression. In our study, a relative fold change of 6.2 in GDNF gene expression by real-time PCR strengthens this association and the fold drop of 5.51 in ASD patients can be considered a diagnostic and therapeutic marker for cognitive deficit in ASD [14].

Comparing our findings to research done by other scholars buttresses these findings and strengthens the view that GDNF may be pivotal in preventing or reversing cognitive deterioration and TD in ASD or related disorders. These findings support previous research showing reduced GDNF activity in individuals with neurodegenerative conditions such as Alzheimer’s disease (AD). High GDNF gene activity has been linked to amyloidogenesis, the production of amyloid protein, which in turn causes amyloid plaques to develop in the brain and worsens neurodegenerative illnesses [15]. GDNF plays a critical role in pathogenic traits, including neuroinflammation and synaptic dysfunction, which are shared by ASD and AD despite being separate illnesses [16]. A vital factor in ASD, a complex disorder, is the decrease in GDNF. There is a wealth of data demonstrating the correlation between lower levels of GDNF and improved cognitive performance.

One limitation of this study is the reliance on GDNF gene expression as a singular biomarker for ASD, which may overlook other key genetic or environmental factors. In addition, GDNF expression was linked to cognitive decline, and direct mechanistic pathways connecting GDNF malfunction and ASD pathology warrant further investigation.

## Conclusions

According to the findings of this study, the GDNF gene shows a great deal of promise as a biomarker for identifying and treating ASD. Early identification of changed GDNF expression may enable prompt and focused therapy interventions, therefore slowing the development of behavioral and cognitive deficits in ASD patients. Furthermore, the distribution of GDNF mRNA in peripheral blood mononuclear cells emphasizes a systemic element, emphasizing the usefulness of peripheral markers in reflecting changes in the central nervous system.
